# The Patient-Specific Implant Created with 3D Printing Technology in Treatment of the Irreparable Radial Head in Chronic Persistent Elbow Instability

**DOI:** 10.1155/2018/9272075

**Published:** 2018-10-23

**Authors:** Suriya Luenam, Arkaphat Kosiyatrakul, Chanon Hansudewechakul, Kantapat Phakdeewisetkul, Boonrat Lohwongwatana, Chedtha Puncreobutr

**Affiliations:** ^1^Department of Orthopaedics, Phramongkutklao Hospital and College of Medicine, Bangkok, Thailand; ^2^Department of Metallurgical Engineering, Faculty of Engineering, Chulalongkorn University, Bangkok, Thailand

## Abstract

Successful treatment of the chronic persistent elbow instability is a challenge for orthopedic surgeons. In this form, it is important to recognize and restore the osseous stabilizer in order to obtain the concentric reduction. In the present report, we describe a case of such injury with irreparable radial head treated with patient-specific radial head prosthesis which was created with 3D printing technology. To our knowledge, this is the first report in clinical use of this kind of prosthesis for the radial head fracture. At a 24-month follow-up visit, the patient was satisfied with the functional outcomes. The Mayo Elbow Performance Index (MEPI) increased from 20 points at the preoperative day to 85 points, and the patient-based Disabilities of the Arm, Shoulder, and Hand (DASH) was reduced from 88.33 points to 28.33 points. Due to the favorable result, replacement of the radial head with the patient-specific implant could be a useful treatment for the irreparable radial head in chronic persistent elbow instability.

## 1. Introduction

The chronic persistent elbow instability which has the association of injury of bony and soft tissue stabilizers is one of the most difficult cases to solve and always attributed to the severely restricting function when the treatment is inadequate [1–3]. This is a real challenging problem even for the most skilled orthopedic surgeons. To obtain a concentric joint reduction and a stable arc of motion, restoration of the osseous stabilizers is a crucial part of the operation when the concomitant fractures were present. Radial head replacement with the metal prosthesis is generally recommended for the irreparable fracture [4, 5].

Several anthropometric studies demonstrated that native radial head has complex anatomy. The radial head is not circular and it does not have a consistently elliptic shape. The radial neck-diaphysis angle and offset from the axis are also variable [6–8]. For this reason, a radial head hemiarthroplasty that precisely replicates the native bone shape would be difficult to achieve [6]. With the advancement of 3D printing technology, it has now become possible to aid in producing a custom prosthesis for individual patient [9, 10]. Based on the symmetry of the human skeleton, we can reverse the 3D images of the normal bone to the contralateral side in order to manufacture the prosthesis which has more natural contact mechanics for the missing part [11]. Using this technique, the tailor-made prostheses for talus, calcaneus, metacarpal, and lunate have been previously reported [12–15]. To our knowledge, no clinical use for such implant has been published by medical journals to date.

In the present report, we describe a case of chronic persistent elbow instability with irreparable radial head treated with patient-specific radial head prosthesis which was created with 3D printing technology.

## 2. Case Report

**Figure 1:**
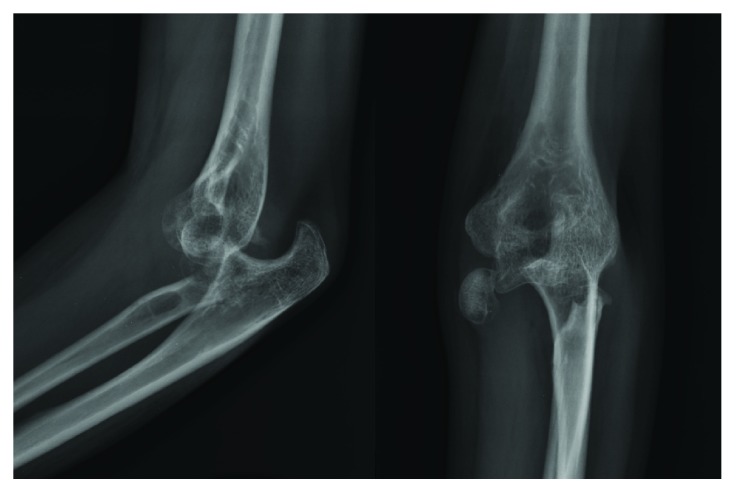
Plain radiographs showing posterior elbow dislocation with displaced radial head.

**Figure 2:**
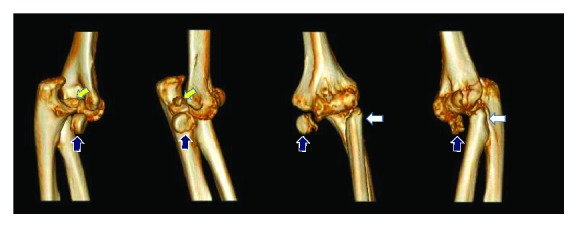
CT scan showing the coronoid tip fracture (yellow arrow) and irreparable radial head fracture with indented articular surface (blue arrow) and deformation of the fracture ends (white arrow).

A 37-year-old female presented to our clinic with restricted movement of the left elbow for a duration of 9 months after history of trauma. The patient was previously treated by the local bonesetter. On physical examination, the elbow was stiff in 40 degrees of flexion. Disruption of the three-point bony relationship between the olecranon tip and medial and lateral epicondyles was revealed. The olecranon tip was prominent posteriorly with tenting of the triceps tendon. No neurologic deficit was observed. The X-rays showed the posterior elbow dislocation with displaced radial head fracture (Figure 1). The CT scan demonstrated the irreparable radial head fracture with indented articular surface as well as the deformation of the fracture ends. A tip fracture of the coronoid process (Reagan-Morrey type I) was noted (Figure 2).

**Figure 3:**
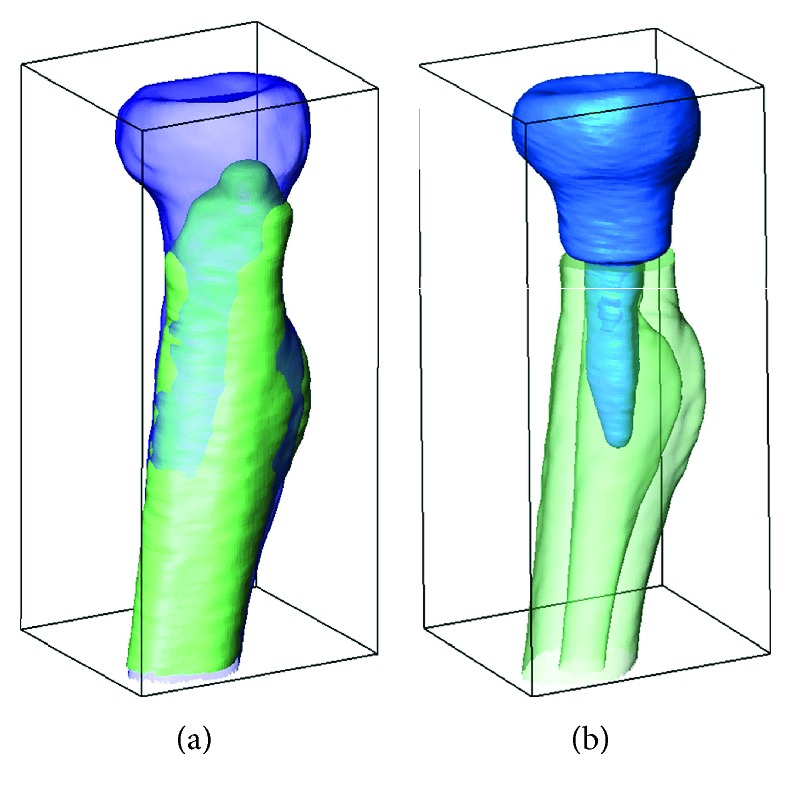
Preoperative planning: (a) 3D registration of the affected side with the mirrored image of the contralateral side and (b) planned level of bone resection and extent of radial head reconstruction.

The surgical treatment with open reduction of the elbow with radial head replacement was planned. In this study, the custom-made anatomical radial head prosthesis was chosen over the commercial radial head prosthesis due to limited availability of the commercial prosthesis in our country. In order to fabricate the patient-specific prosthesis, a high-resolution CT scanning (Philips Brilliance 64 CT scanner, Cleveland, OH; voxel size 0.45 × 0.45 × 0.45 mm, 120 kV, 150 mAs, pitch 0.6) of both elbows was performed and reconstructed into 3D images. Subsequently, 3D image of the affected side was aligned with the mirrored 3D image of the contralateral bone by registration of the radial tuberosity and diaphysis to identify the correct anatomic profile of the reconstructed part (Figure 3(a)). The aligned 3D images were then processed through image processing techniques and computer-aided design (CAD) to construct a 3D prosthesis model. With this technique, accurate preoperative planning for the position of additional bone resection and extent of radial neck restoration can be established (Figure 3(b)). The stem configuration of the prosthesis was designed in conforming to the alignment of the intramedullary canal while the stem length was determined to achieve a cantilever quotient of 0.5. To aid in filling of bone cement, a free space of 1 mm between the prosthesis stem and the bone was also maintained.

**Figure 4:**
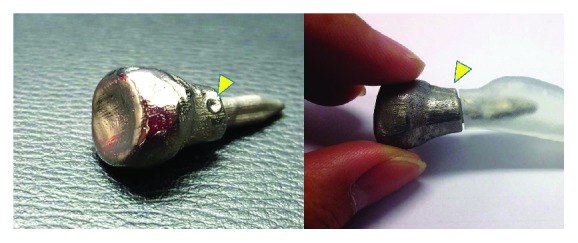
Photographs of titanium prosthesis with impressed mark (yellow arrowhead) to guide the rotation alignment.

**Figure 5:**
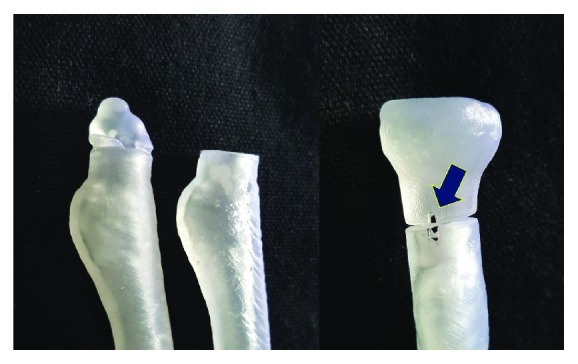
Photographs of 3D resin models of the proximal radius representing the cutting line and location of the impressed (blue arrow) for intraoperative guidance.

Once the reconstruction of computerized radial head prosthesis was completed, a STL file was generated for fabrication of resin-customized implant using the stereolithography (SLA) technique. This 3D-printed resin model was directly used as a master pattern for investment casting. The resin model was first embedded with a high-temperature resistance ceramic. Subsequently, it was heated up to a temperature range of 450°C–1000°C to obtain a ceramic mold for titanium casting. Finally, the customized titanium implant was produced by investment casting in a clean environment. The hand polishing was performed to smooth the prosthetic surface. The roughness of the final polished prosthesis is in a range of 20 ± 10 micrometers. The complete manufacture of the prosthesis from the original CT took a total of 10 days. Note that the impressed mark in line with the radial tuberosity was specifically designed on the surface of the prosthesis to guide for the rotation alignment (Figure 4). The 3D resin models of the proximal radius representing the cutting line were also fabricated using the 3D printing technique for intraoperative guidance (Figure 5).

The patient was operated under general anesthesia and was placed on the ordinary surgical table in supine. The injured arm rested on the support. A tourniquet was placed proximally on the arm.

**Figure 6:**
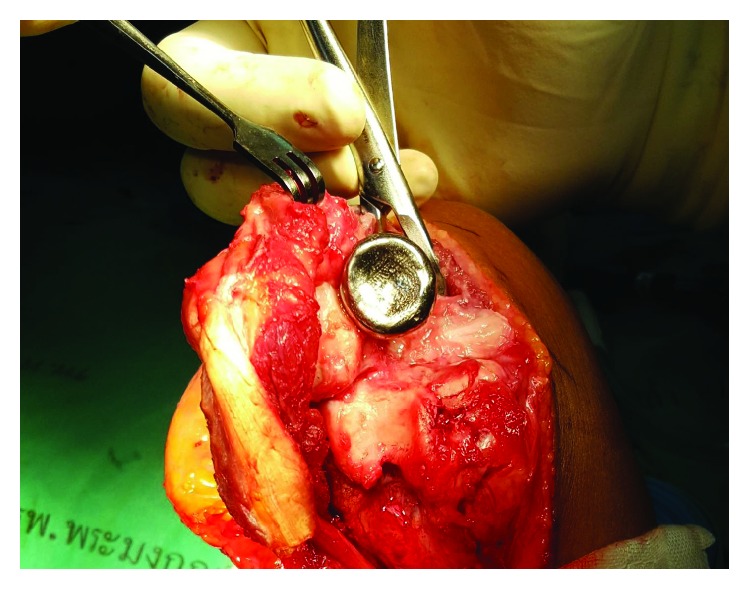
Intraoperative photograph showing the congruity of the titanium prosthesis with the sigmoid notch.

The incision was made on the posterior aspect of the elbow, beginning at the midline 7 cm proximal to the olecranon, curve the incision laterally around the olecranon and continue farther distally along the line of proximal ulna for 7 cm. The ulnar nerve and posterior interosseous nerve were identified and isolated with elastic sling. The shortened triceps bound down by fibrous tissue to the humerus was incised and lengthened using a Speed V-Y muscleplasty technique [16]. The contracted capsule and collateral ligaments were cut. Dense fibrous tissue filled up the olecranon; the coronoid fossae was carefully excised to avoid peeling off of the underlying cartilage. A large articular bone defect on the medial trochlea was observed. The radial head fragment and the fracture ends were exposed. Severe cartilage damage of the radial head with the metaphyseal bone loss was noted. The radial neck was resected with the microsagittal saw according to the preoperative planning. Cancellous bone in the intramedullary canal was removed using the bony curette, and the canal was irrigated with saline solution. A bone chip harvested from the radial head had been inserted into the canal as a cement restrictor. An acrylic cement (Palacos® radiopaque bone cement 1 × 40 g Single, 40.8 g methyl acrylate copolymer, 20 ml methyl methacrylate monomer 0.5 g gentamicin, Zimmer Dover, OH) was mixed and applied into the canal. The radial head prosthesis was introduced into the canal with the impressed mark aligned with radial tuberosity until the prosthetic neck fully seated on the cutting cortex (Figure 6). Excess cement was cleared from the prosthesis-bone junction. The collateral ligament and triceps aponeurosis were repaired. Concentric reduction of radioulnar, radiocapitellar, and ulnohumeral joints through the entire range of motion was assessed under clinical examination and fluoroscopy. Fixation of the coronoid fracture was not performed as the elbow stability though a functional range of motion was sufficiently restored with the radial head replacement and collateral ligament repair.

Postoperatively, the arm was immobilized in a posterior splint at 90°. Active-assisted range-of-motion exercises were initiated in 10 days after the surgery. We did not use any medications or irradiation as prophylaxis against heterotopic ossification.

**Figure 7:**
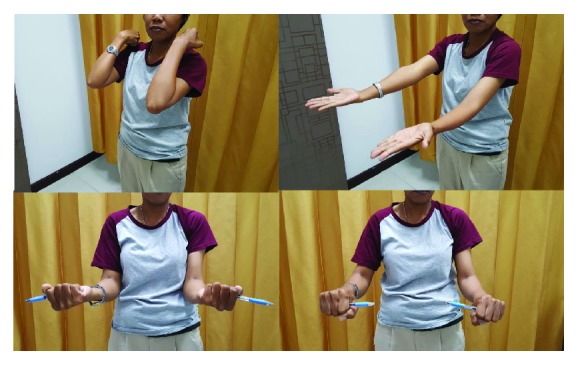
Postoperative range of motion at 24 months after the surgery.

**Figure 8:**
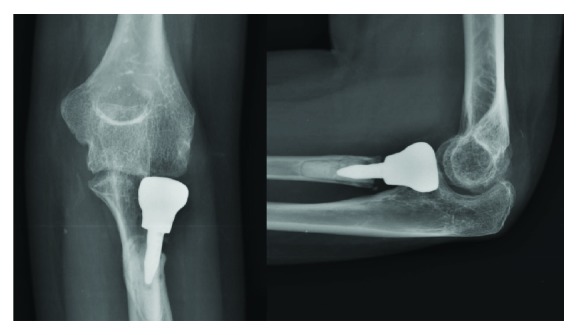
Postoperative plain radiographs at the last follow-up.

At the latest follow-up, 24 months after surgery, the elbow extension was 28°, flexion was 145°, pronation was 80°, and supination was 90° (Figure 7). Hand grip power of the injured side averaged 95.4% of the normal side (27.6 kg for injured arm and 28.3 for normal arm). The Mayo Elbow Performance Index (MEPI) increased from 20 points at the preoperative day to 85 points, and the patient-based Disabilities of the Arm, Shoulder, and Hand (DASH) was reduced from 88.33 points to 28.33 points. The patient was satisfied with the cosmetic and functional outcomes. The last follow-up X-rays demonstrated the concentric elbow joint, but the narrowing of the radiocapitellar joint space is noted. The radiolucency around the bone-cement interface was apparent, but no progression was seen compared with the X-rays done at 12 months postoperatively. The proximal bone resorption at the radial neck and capitellar osteopenia were observed (Figure 8).

## 3. Discussion

Radial head replacement is essential in treatment of the chronic persistent elbow dislocation associated with the irreparable radial head fracture [17]. In the present case, the custom prosthesis manufactured to mimic the original radial head using 3D printing technology had been used. To our knowledge, this is the first report in clinical use of this kind of prosthesis.

We have chosen titanium as an implant material used for the prosthesis in this case because of its high biocompatibility, good mechanochemical properties, and lightweight nature. However, the titanium casting process is more technically demanding than that of other materials such as stainless steel and cobalt chrome [14]. The stem was designed to be cemented instead of the press-fit because the amount of intraoperative bone removal in each part of the inner cortex for the canal preparation is unpredictable with the 3D CT model. The error in planning of the press-fit stem shape and diameter may result in the incorrect alignment, inadequate stem stability, or intraoperative fracture. In this case, the prosthetic alignment was relied on the fit of the edge of the prosthetic neck on the osteotomy cut. The undersized stem was created to leave a space for the cement mantle and allow fine adjustment of the prosthetic alignment.

The surgical outcome in treatment of the chronic persistent elbow instability was previously reported by many authors [2, 4, 18]. Most of the reports are case series which combined the dislocation with and without associated fractures in the review. The suboptimal results with potential treatment failure were noted when associated radial head fracture was presented [4, 19]. Residual range of motion deficit and flexion contracture is commonly seen following the treatment [2, 4, 18, 19]. In the present case, the arc of motion was greater than 90 degrees, the flexion contracture was less than 30 degrees, and the elbow was painless and stable postoperatively. This was classified as a good clinical result according to the grading system described by Krishnamoorthy et al. [20].

The postoperative radiographs done at 24 months postoperatively demonstrated the concentric elbow joint but the narrowing of the radiocapitellar joint, capitellar osteopenia, proximal bone resorption at the radial neck, and radiolucency of the bone-cement junction were found. The narrowing of the radiocapitellar joint suggesting the development of capitellar cartilage erosion and osteoarthritic change probably resulted from the preexisting poor cartilage condition, the metal prosthetic surface, and implant malposition. An irreversible degenerative change of the hyaline cartilage always occurs in chronic dislocation because of the long-term impairment of the cartilage metabolism and synovial fluid function [21]. According to the previous animal study, the metal surface of prosthesis articulating on the cartilage is detrimental to the cartilage [22]. As our prosthesis is homemade, an inadequate polishing of the implant surface may cause cartilage abrasion. The malposition of prosthesis may result in maltracking and capitellar erosion. Although not well visualized in the present case, the malposition of the prosthesis could occur from the nonanatomical insertion in up to 50% of the prostheses in an in vitro study [23]. The capitellar osteopenia found in the postoperative radiographs may be related to the abnormal load pattern after implantation of the prosthesis. Based on the current literature, this condition occurred in both overstuffing and understuffing of the prosthesis [24, 25]. The proximal bone resorption at the radial neck and radiolucency of the bone cement junction were also found in the case series of bipolar and monoblock radial head implant with cemented stem [26, 27]. Burkhart et al. suggested that these findings resulted from the insufficient cementing techniques [28]. Further follow-up is needed to verify whether they will eventually lead to failure of the implant or not. According to these unfavorable radiographic findings, further development of the stem design and cementation technique, polishing and buffering processes for smoothing a prosthetic surface, and intraoperative guide for accurate insertion may be required to improve the long-term performance.

In order to generate the reverse-engineered patient-specific implants, the bilateral arm CT scan is needed which made the treatment cost higher. The CT scan cost is variable in different regions around the world. In some countries, the high price of CT may make the surgeons hesitate to use such implant. The additional cost of the manufacturing process including the computer-aided design, printing of resin model, and metal casting is also a concern [29]. Surprisingly, the pilot cadaveric study in the production of the 3D-printed custom radial head metal implant revealed an inexpensiveness in total cost [30]. It is rather complicated to calculate the exact cost for the manufacturing of the prosthesis in our case, since the authors performed this work (data processing, resin 3D printing, and titanium casting) ourselves by using the facilities at our academic institutes. In the present case, complete production of the prosthesis from the original CT took 10 days. The same amount period of processing time was described in the pilot study [30]. The development of 3D printing technology in creating the prostheses directly from the CT model could reduce the processing time which may be suitable to be used in acute setting as well [9, 10].

In conclusion, a patient-specific radial head implant created with 3D printing technology appears to provide satisfactory functional outcome. This could be a useful treatment for the irreparable radial head in chronic persistent elbow instability.
